# Genetic characteristics associated with the virulence of porcine epidemic diarrhea virus (PEDV) with a naturally occurring truncated ORF3 gene

**DOI:** 10.1186/s13567-024-01384-w

**Published:** 2024-09-27

**Authors:** Ying Lu, Weijian Huang, Zhengpu Lu, Deping Zeng, Kechen Yu, Jiaguo Bai, Qiuying Qin, Meijin Long, Yifeng Qin, Ying Chen, Zuzhang Wei, Kang Ouyang

**Affiliations:** 1 College of Animal Science and Technology, Guangxi University, Nanning, China; 2 Guangxi Zhuang Autonomous Region Engineering Research Center of Veterinary Biologics, Nanning, China; 3 Guangxi Key Laboratory of Animal Reproduction, Breeding and Disease Control, Nanning, China

**Keywords:** Porcine epidemic diarrhea virus, virus attenuation, ORF3, mutation, pathogenicity

## Abstract

Porcine epidemic diarrhea virus (PEDV) has emerged in American countries, and it has reemerged in Asia and Europe, causing significant economic losses to the pig industry worldwide. In the present study, the 17GXCZ-1ORF3d strain, which has a naturally large deletion at the 172–554 bp position of the ORF3 gene, together with the 17GXCZ-1ORF3c strain, was serially propagated in Vero cells for up to 120 passages. The adaptability of the two strains gradually increased through serial passages in vitro. Genetic variation analysis of the variants of the two strains from different generations revealed that the naturally truncated ORF3 gene in the 17GXCZ-1ORF3d variants was stably inherited. Furthermore, the survival, viral shedding and histopathological lesions following inoculation of piglets demonstrated that the virulence of 17GXCZ-1ORF3d-P120 was significantly attenuated. These results indicate that the naturally truncated ORF3 gene may accelerate the attenuation of virulence and is involved in PEDV virulence together with mutations in other structural genes. Importantly, immunization of sows with G2b 17GXCZ-1ORF3d-P120 increased PEDV-specific IgG and IgA antibody levels in piglets and conferred partial passive protection against heterologous G2a PEDV strains. Our findings suggest that an attenuated strain with a truncated ORF3 gene may be a promising candidate for protection against PEDV.

**Supplementary Information:**

The online version contains supplementary material available at 10.1186/s13567-024-01384-w.

## Introduction

Porcine epidemic diarrhea (PED), which is characterized by severe watery diarrhea, vomiting, dehydration and a high mortality rate in piglets, results in significant economic losses to the swine industry worldwide [1]. Porcine epidemic diarrhea virus (PEDV) was first reported in England in the late 1970s, and subsequently, the G1a subgroup of PEDV strains became widespread in Europe and Asia [2, 3]. At the end of 2010, a large outbreak of PED in the G2 group occurred in China, despite most of the sows had been immunized with a PEDV inactivated vaccine derived from the CV777 strain, which belongs to the G1 subgroup [4]. A highly virulent PEDV G2b subgroup subsequently emerged in the USA in 2013 [5], and a new variant called subgroup S-INDEL strain (G1b) has expanded to additional states in the USA and Canada [6]. The emergence and re-emergence of PED represents a major threat worldwide and is recognized as one of the most economically important diseases in the swine industry [7].

PEDV is an enveloped, single-stranded, positive-sense RNA virus that belongs to the family *Coronaviridae* and genus *Alphacoronavirus*. The viral genome is approximately 28 kb in length and has seven open reading frames (ORFs), each of which encodes specific proteins. There are two nonstructural proteins (ORF1a and ORF1b) and a spike (S), an accessory (ORF3), an envelope (E), a membrane (M) and a nucleocapsid (N) protein [8]. The S protein, one of the proteins expressed by the ORFs, is crucial for the attachment of viral particles to host cell receptors [9]. PEDV is divided into groups 1 (G1) and 2 (G2) on the basis of the S gene, and the ORF3 gene presents a comparable level of genetic diversity [9]. The S and ORF3 genes are valuable tools for molecular epidemiological studies of PEDV infections [10].

ORF3, the only accessory protein of PEDV, plays an important role in viral replication as well as pathogenicity [11]. Generally, mutations and deletions in the ORF3 gene are frequently associated with cell adaptation and attenuated virulence, and nucleotide deletions can lead to premature termination of ORF3 translation [12, 13]. The PEDV 17GXCZ-1ORF3d strain obtained previously in our laboratory, with a naturally truncated ORF3 gene at 172–554 bp, can cause severe diarrhea in piglets, resulting in high mortality [14]. PEDV carrying the naturally truncated ORF3 gene subsequently exhibited high virulence in Henan pig farms in China [15].

Currently, immunization, along with stringent biosecurity measures, is crucial in the prevention of PED. Since 2010, due to the high variability of PEDV, there has been a lack of efficient and safe vaccines, which poses a challenge for the prevention and control of new epidemics [16, 17]. Live attenuated vaccine candidate strains were obtained by serial passaging of PEDV strains in Vero cells [18–23]. In the present study, 17GXCZ-1ORF3d, with a naturally truncated ORF3 gene, was attenuated by being serially passaged in vitro, and the genetic characteristics associated with the pathogenicity of the different generations were further investigated. Moreover, the “live + inactivated” immunization strategy is currently commonly used in large-scale swine farms in China [24]. To evaluate the efficacy of passive protection against heterologous G2a PEDV, we immunized sows with the live G2b PEDV 17GXCZ-1ORF3d-P120 vaccine candidate combined with a commercial inactivated G2b vaccine.

## Materials and methods

### Cells, viruses and antibodies

Vero cells (African green monkey kidney cells) were maintained in high-glucose DMEM (Life Technologies, Carlsbad, CA, USA) supplemented with antibiotics and 10% fetal bovine serum (Biological Industries, Kibbutz Beit Haemek, Israel) at 37 ℃ in a 5% CO_2_ incubator. The PEDV G2 strains 17GXCZ-1ORF3d and 17GXCZ-1ORF3c (GenBank accession nos. MT547179 and MT547180, respectively) were previously isolated from Vero cells in our laboratory. A mouse anti-PEDV spike protein monoclonal antibody (Median, Chuncheon, Korea; diluted 1:500), an Alexa Fluor™ 488-conjugated goat anti-mouse IgG (H + L) antibody (Invitrogen, CA, MSA; diluted 1:4000) and 4′6-diamidino-2-phenylindole (DAPI; Beyotime) were purchased.

### Serial passage of strains 17GXCZ-1ORF3d and 17GXCZ-1ORF3c in Vero cells

Vero cells were cultured in T25 cell culture flasks and grown to 90% confluence. The 17GXCZ-1ORF3d and 17GXCZ-1ORF3c strain stocks at the 15^th^ passage, 17GXCZ-1ORF3d-P15 and 17GXCZ-1ORF3c-P15, respectively, were inoculated with Vero cells. After shaking every 15 min for 1 h, the viral suspension was discarded, the cell culture flasks were washed twice with PBS, and 5 mL of maintenance medium containing 15 μg/mL trypsin was added. When 80% of the cells had developed a visible cytopathic effect (CPE), the infected cells were lysed by freeze‒thawing and centrifuged at 4000 × *g* for 1 min at 4 °C to harvest 17GXCZ-1ORF3d and 17GXCZ-1ORF3c for the next passages. These viral stocks were then used to inoculate monolayers of Vero cells grown in T25 cell culture flasks. Further propagation was continued in the same way until the 120^th^ passage (17GXCZ-1ORF3d-P120 and 17GXCZ-1ORF3c-P120, respectively).

### Plaque assay

Vero cells in six-well plates were inoculated with 200 μL of tenfold serially diluted PEDV. After 1 h of incubation at 37 °C, the cell monolayers were washed with phosphate-buffered saline (PBS) and overlaid with 1% low-melting point agarose with 15 μg/mL trypsin. After the gel overlay solidified, the plates were inverted and placed in an incubator at 37 ℃ with 5% CO_2_. At 3–4 days post-infection (dpi), plaques were selected for infection, and they were visualized by using crystal violet staining. Ten plaques were randomly selected, and their diameters were measured using a ruler with ImageJ 1.8.0 software.

### Immunofluorescence assay (IFA)

Vero cells were grown to 70–80% confluence in 96-well plates and inoculated with the PEDV strains 17GXCZ-1ORF3d and 17GXCZ-1ORF3c. Medium alone was used as a control. At 72 h post-infection (hpi), the cells were fixed with cold formaldehyde and blocked with PBS containing 1% bovine serum albumin (BSA). After blocking, the cells were incubated with an anti-PEDV spike protein monoclonal antibody (Median, Chuncheon, Korea; diluted 1:500) for 2 h at 37 ℃. The cells were washed with PBS three times and then incubated with an Alexa Fluor™ 488-conjugated goat anti-mouse IgG (H + L) antibody (Invitrogen, CA, MSA; diluted 1:4000) for 1 h. Finally, the cells were washed and then visualized by fluorescence microscopy.

### Genome sequencing

Total RNA from the P30, P60, P90 and P120 PEDV strains of 17GXCZ-1ORF3d and 17GXCZ-1ORF3c strains was extracted using a viral DNA/RNA kit (Axygen Scientific, Union City, CA, USA) and transcribed into cDNA using oligo dTs, dNTP mix and M-MLV reverse transcriptase reagent (TaKaRa, Dalian, China), respectively. To obtain the PEDV structural gene and ORF3 gene sequences, the primers used in the previous study were amplified separately [14]. The products were purified and cloned and inserted into a pMD-18 T vector (TaKaRa, Dalian, China), and the resulting sequences were determined by the Beijing Genomics Institute (Guangzhou, China).

### Multiple alignments and phylogenetic analyses

The amino acid sequences of the S proteins of these strains were also aligned via the Clustal W method in MegAlign. The S protein, S–N gene nucleotides and ORF3 protein of different generations of 17GXCZ-1ORF3d and 17GXCZ-1ORF3c strains were compared with those of 107 reference strains (Additional file 1) in GenBank to construct a phylogenetic tree using the neighbour-joining (NJ) method of MEGA-X software, followed by bootstrap analysis of 1000 replicates to determine the percentage reliability value of each internal node of the tree. The resulting tree was visualized using iTOLv.5 (Interactive Tree of Life).

### Pathogenicity evaluation of the 17GXCZ-1ORF3d and 17GXCZ-1ORF3c variants

Twenty-eight 5-day-old conventional piglets were purchased from a commercial pig farm with no PEDV vaccination program and no history of PED. In accordance with reported pathogenicity studies on PEDV [22, 25], all piglets were diagnosed as negative for PEDV, transmissible gastroenteritis virus (TGEV), rotavirus (PoRV), porcine deltacoronavirus (PDCoV), classical swine fever virus (CSFV), porcine reproductive and respiratory syndrome virus (PRRSV) and pseudorabies virus (PRV) by RT-PCR. One piglet died from stress caused by adapting to a new housing environment, and the remaining twenty-seven piglets were divided into seven groups of three or four animals each. Piglets were randomly assigned to the seven experimental groups, and they were housed in separate rooms. In accordance with a previous study [26], the piglets were artificially fed milk replacer every 4–6 h. Four piglets from each group were inoculated orally with P15 (Group A), P90 (Group B) and P120 (Group C) of 17GXCZ-1ORF3d and P15 (Group D), P90 (Group E) and P120 (Group F) of 17GXCZ-1ORF3c at a dose of 6 log_10_ PFU/mL. The mock group (Group G), which included three piglets, was inoculated with the same volume of cell culture medium. During the experiment, observations of clinical signs and collection of rectal swabs were conducted as described. The shedding of fecal viral RNA was determined by RT-quantitative PCR (RT-qPCR) as described previously [14]. The piglets were necropsied upon death, and the surviving piglets in the challenged and mock groups were euthanized at 14 dpi. Piglets were euthanized according to a humanitarian endpoint. The procedures for necropsy and tissue sampling, as well as haematoxylin & eosin (H&E) and immunohistochemistry (IHC) staining, were conducted as described previously [14], and different tissues were collected for viral load detection via RT-qPCR.

### Immunogenicity evaluations of 17GXCZ-1ORF3d-P120 based vaccine candidates

Ten commercial crossbred sows with the same parity and expected farrowing date were randomly divided into two groups (5 sows per group). The live G2b 17GXCZ-1ORF3d-P120 vaccine candidate (2 × 10^6^ PFU per sow) or PBS was injected into the necks of 5 sows 4 weeks before parturition. The immunized sows also received a G2b commercial inactivated vaccine 2 weeks later. All sows were monitored daily for clinical changes and were allowed to farrow naturally for the duration of the study. After being allowed to suckle for 7 days, 5 piglets were randomly selected from each sow (a total of 25 piglets per group), named the 17GXCZ-1ORF3d-P120 group and challenged control group, respectively, and were orally dosed with 2 mL of the G2a PEDV homogenized intestinal tissue. Clinical signs of piglet diarrhea were monitored daily throughout the study, fecal samples were collected at 2, 5, 9, 13 and 17 days post-challenge (dpc) after oral administration of the virus, and viral shedding was assessed by RT-qPCR. Serum samples were collected at 2, 5, 9, 13 and 17 dpc, and PEDV-specific IgG and IgA antibodies were detected by ELISA according to the manufacturer’s instructions (Biostone, Dallas, Texas, USA). Six piglets from each group were euthanized at 17 dpc for postmortem examination, and different tissues were collected for viral load assessment by RT-qPCR. The survival rates of all the piglets were calculated.

### Statistical analysis

All the values are expressed as the means ± standard errors of the means (SEMs). Statistical analysis was performed by Student’s *t* test using GraphPad Prism 8 (GraphPad, La Jolla, CA, USA). A value of *P* < 0.05 was considered statistically significant, *P* < 0.01 was considered highly significant, and *P* < 0.001 was considered extremely significant.

## Results

### Biological characteristics of the 17GXCZ-1ORF3d and 17GXCZ-1ORF3c strains after serial passages in vitro

**Figure 1:**
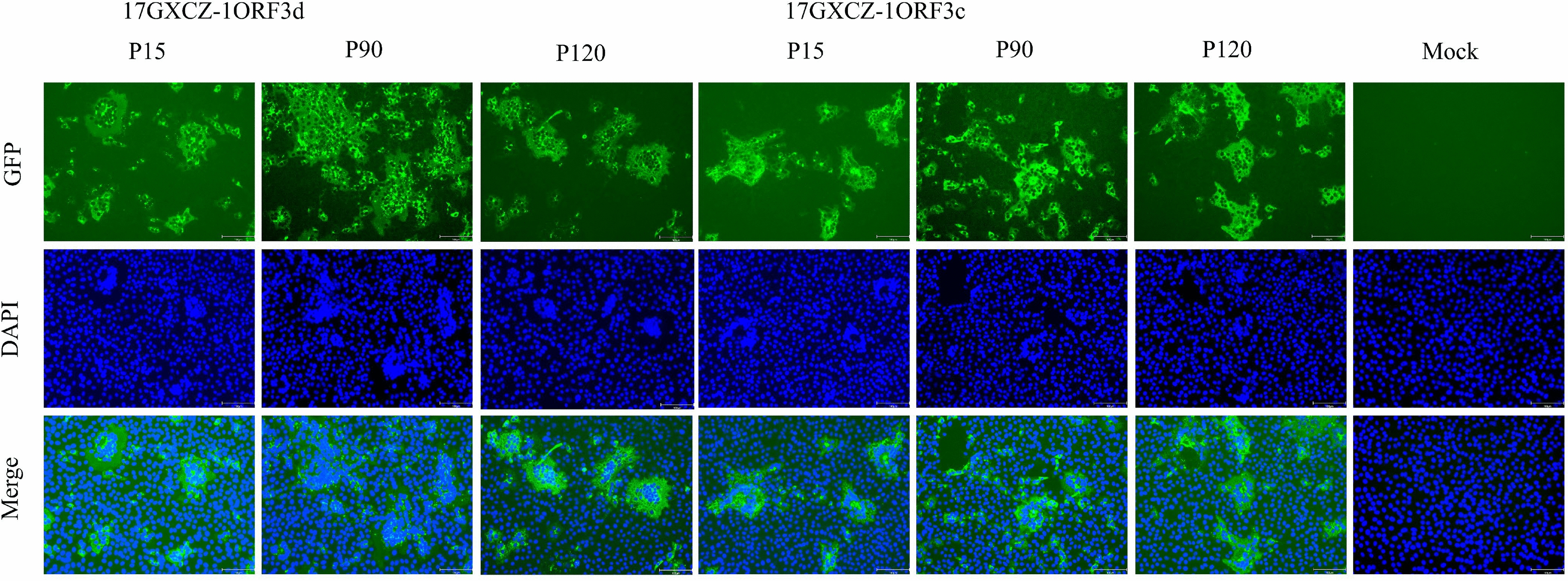
**Immunofluorescence of PEDV S (green) detected in Vero cells infected with the 17GXCZ-1ORF3d and 17GXCZ-1ORF3c variants**. For immunostaining, infected cells were fixed at 72 hpi and incubated with an anti-PEDV spike protein monoclonal antibody followed by incubation with an Alexa Fluor 488-conjugated goat anti-mouse antibody, after which nuclear staining was performed with DAPI. Finally, the cells were examined under a fluorescence microscope at 200 × magnification.

**Figure 2:**
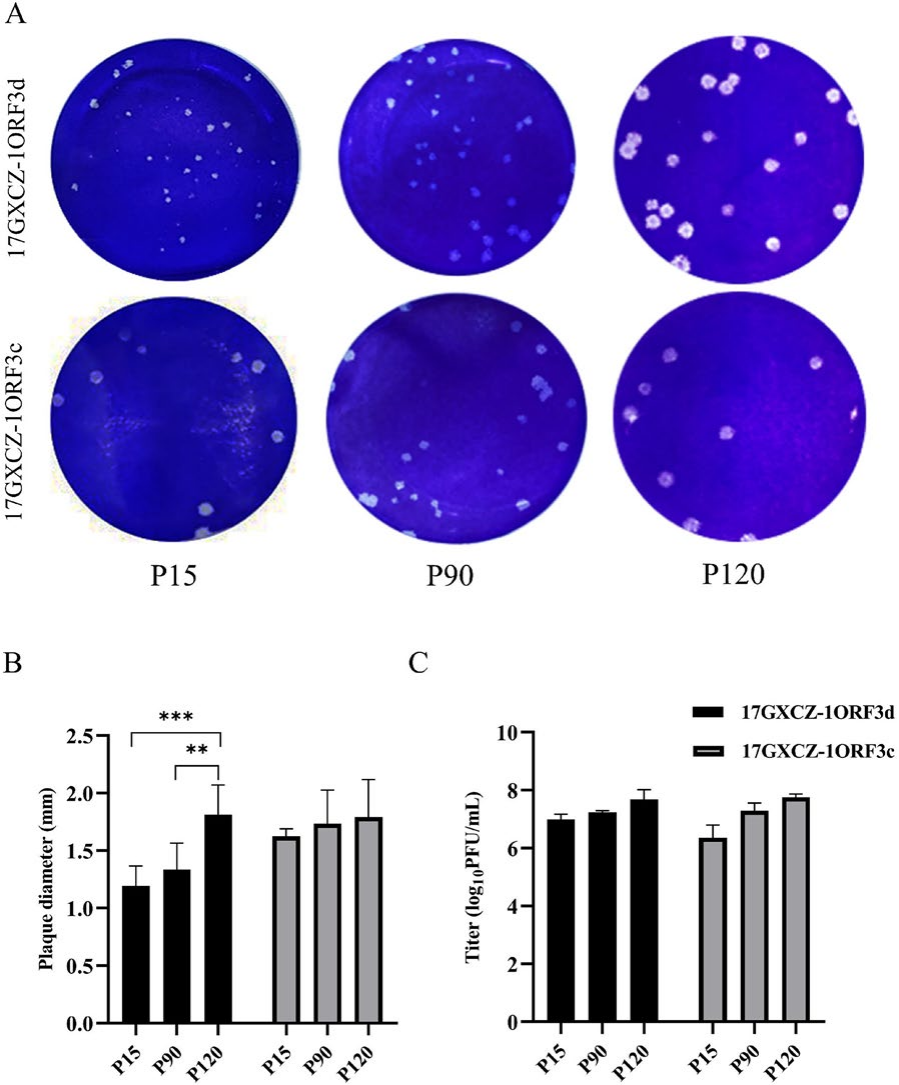
**Biological characteristics of PEDV17GXCZ-1ORF3d and 17GXCZ-1ORF3c variants during serial passaging in vitro**. **A** Plaque formation assay in different generations of the PEDV 17GXCZ-1ORF3d and 17GXCZ-1ORF3c variant strains. Vero cell monolayers were inoculated with tenfold serial dilutions of different generations of variants of PEDV strains, namely, 17GXCZ-1ORF3d and 17GXCZ-1ORF3c. After 1 h of incubation, the cells were covered with 1% agarose. The plaques were stained with crystal violet at 3–4 dpi. **B** Plaque diameters of different generations of 17GXCZ-1ORF3d and 17GXCZ-1ORF3c variants. The diameters of ten randomly selected patches were measured with a ruler in ImageJ 1.8.0 software. The asterisks indicate that 17GXCZ-1ORF3d-P90 and 17GXCZ-1ORF3d-P120 were significantly different from 17GXCZ-1ORF3d-P15 (**P < 0.01 and ***P < 0.001). **C** Viral titres of different generations of 17GXCZ-1ORF3d and 17GXCZ-1ORF3c variants in Vero cells. Vero cells were inoculated with 17GXCZ-1ORF3d or 17GXCZ-1ORF3c variants at an MOI of 0.01. The supernatants were collected at 48 hpi and measured via plaque assay for virus titration.

We initially conducted in vitro serial passaging, and different generations of the variants were selected for identification by IFA. Virus propagation was confirmed by detecting PEDV antigens by IFA using a PEDV S protein-specific monoclonal antibody. The results revealed that the attached fluorophore could be detected in Vero cells infected with different generations of the variants, whereas no fluorophore was detected in the mock group (Figure 1). The plaque sizes and morphologies of the 17GXCZ-1ORF3d variants were compared with those of the 17GXCZ-1ORF3c variants (Figure 2A). The mean plaque diameter of 17GXCZ-1ORF3d-P120 (1.81 mm) was significantly larger than those of 17GXCZ-1ORF3d-P90 and 17GXCZ-1ORF3d-P15 (1.33 and 1.19 mm; *P* < 0.01 and 0.001, respectively; Figure 2B). To investigate the in vitro phenotypic characteristics of serially passaged PEDV strains, the viral titres of different generations were determined via plaque assays. The titre for 17GXCZ-1ORF3d-P15 was 10^6.99^ PFU/mL, and those of P90 and P120 of this strain were 10^7.24^ and 10^7.68^ PFU/mL, respectively. The 17GXCZ-1ORF3c variant titres ranged from 10^6.36^ to 10^7.75^ PFU/mL (Figure 2C). These results suggested that the ability of the 17GXCZ-1ORF3d and 17GXCZ-1ORF3c variants to adapt to Vero cells in culture gradually increased during serial passages in vitro.

### Amino acid changes in the ORFs during serial passaging

The S–N nt sequences of the 17GXCZ-1ORF3d and 17GXCZ-1ORF3c variants were determined to investigate their genomic changes during in vitro serial passages in Vero cells. The genomic sequences of these variants (P30, P60, P90 and P120) were compared with P15 using ClustalW in MegAlign software. The results revealed that, relative to 17GXCZ-1ORF3d-P15, the other passaged variants (P30, P60, P90 and P120) had 7, 8, 11 and 13 aa changes, respectively. The 17GXCZ-1ORF3d-P120 strain is relatively conserved in the ORF3 and E regions, with no aa changes detected in either gene. However, there were different amounts of aa changes in S, M and N, which accounted for 0.51, 1.32 and 0.68% of their total aa, respectively (Table 1). 17GXCZ-1ORF3d-P120 had 7 aa changes (S27L, P467S, E636K, Y652C, S887R, G888R and A969S) in the S protein, and among them, the E636K mutation was located in the core neutralizing epitope (COE). There were 3 aa changes in the M and N proteins. In contrast, variants (P30, P60, P90 and P120) in 17GXCZ-1ORF3c had 5, 12, 20 and 18 aa changes, respectively. Among them, there were 9, 3, 2, 1 and 3 aa changes in S, ORF3, E, M and N in 17GXCZ-1ORF3c-P120, respectively. The 17GXCZ-1ORF3c-P120 strain presented 9 aa changes (A119V, A286S, D448A, V689A, Y764F, S887R, A969S, L981P and N1260D) in the S protein and 3 aa changes (F76S, I144V and E188D) in the ORF3 protein. In addition, the F76S mutation is located in transmembrane (TM) domain 2 of the ORF3 protein. Importantly, two mutations, S887R and A969S, in the S protein were detected in both the 17GXCZ-1ORF3d-P120 and the 17GXCZ-1ORF3c-P120 strains. Details on the aa changes among the 17GXCZ-1ORF3d and 17GXCZ-1ORF3c variants are summarized in Table 2. These results revealed genomic changes in the 17GXCZ-1ORF3d and 17GXCZ-1ORF3c variants during serial passaging in vitro.

**Table 1:** **Statistics of the number of a changes and rates of change in the corresponding proteins at different passages of the 17GXCZ-1ORF3d and 17GXCZ-1ORF3c variants**

ORFs	17GXCZ-1ORF3d					17GXCZ-1ORF3c				
	Size (aa)	P30	P60	P90	P120	Size (aa)	P30	P60	P90	P120
S	1386	3 (0.22)	5 (0.36)	8 (0.58)	7 (0.51)	1386	2 (0.14)	7 (0.51)	12 (0.87)	9 (0.65)
ORF3	89	0 (0)	0 (0)	0 (0)	0 (0)	225	1 (0.44)	3 (1.33)	2 (0.89)	3 (1.33)
E	77	2 (2.6)	0 (0)	0 (0)	0 (0)	77	0 (0)	0 (0)	2 (2.60)	2 (2.60)
M	227	2 (0.88)	1 (0.44)	1 (0.44)	3 (1.32)	227	0 (0)	0 (0)	1 (0.44)	1 (0.44)
N	442	0 (0)	2 (0.45)	2 (0.45)	3 (0.68)	442	2 (0.45)	2 (0.45)	3 (0.68)	3 (0.68)
Total		7	8	11	13		5	12	20	18

The number of aa changes and change rates of the corresponding proteins (%) are indicated in parentheses. Amino acid position numbering is based on the sequences of the 17GXCZ-1ORF3d-P15 and 17GXCZ-1ORF3c-P15 strains.

**Table 2:** **Amino acid changes during serial passaging**

Gene	Amino acid position	17GXCZ-1ORF3d						Gene	Amino acid position	17GXCZ-1ORF3c					
		P15	P30	P60	P90	P120				P15	P30	P60		P90	P120
S	27	S	S	S	L	L		S	119	A	A	A		A	V
	158	M	M	I	M	M			156	A	A	A		V	A
	467	P	P	P	H	S			286	A	A	A		A	S
	487	I	Y	I	I	I			300	N	N	N		K	N
	488	S	L	S	S	S			306	V	V	V		I	V
	537	N	N	S	N	N			426	F	F	L		F	F
	574	F	F	F	L	F			433	D	N	D		D	D
	636	E	E	K	K	K			448	D	D	A		A	A
	652	Y	Y	Y	Y	C			476	V	V	V		A	V
	665	L	L	L	P	L			519	A	A	A		V	A
	887	S	S	S	S	R			689	V	V	V		V	A
	888	G	G	R	R	R			764	Y	Y	F		F	F
	969	A	A	S	S	S			830	C	C	C		R	C
	1156	T	T	T	A	T			887	S	S	R		R	R
	1243	D	E	D	D	D			969	A	A	S		S	S
ORF3	–	–	–	–	–	–			981	L	L	L		L	P
E	51	I	T	I	I	I			1037	K	K	E		E	K
	62	S	F	S	S	S			1260	N	N	D		D	D
M	12	I	I	I	I	V			1263	G	C	G		G	G
	28	I	T	T	T	T		ORF3	76	F	S	S		S	S
	54	I	V	I	I	I			144	I	I	I		I	V
	163	S	S	S	S	T			158	N	N	S		N	N
N	106	E	E	E	G	E			188	E	E	D		D	D
	179	G	G	G	E	G		E	12	V	V	V		A	V
	205	K	K	K	K	R			50	Y	Y	Y		C	C
	261	P	P	P	P	S			54	G	G	G		G	R
	287	G	G	A	G	G		M	95	Y	Y	Y		H	H
	288	E	E	G	E	E		N	157	N	H	H		H	H
	424	D	D	D	D	E			216	M	M	M		V	M
									223	V	M	M		M	M
									286	K	K	K		K	R

### Genome sequence analysis and phylogenetic characterization of 17GXCZ-1ORF3d and 17GXCZ-1ORF3c variants

**Figure 3:**
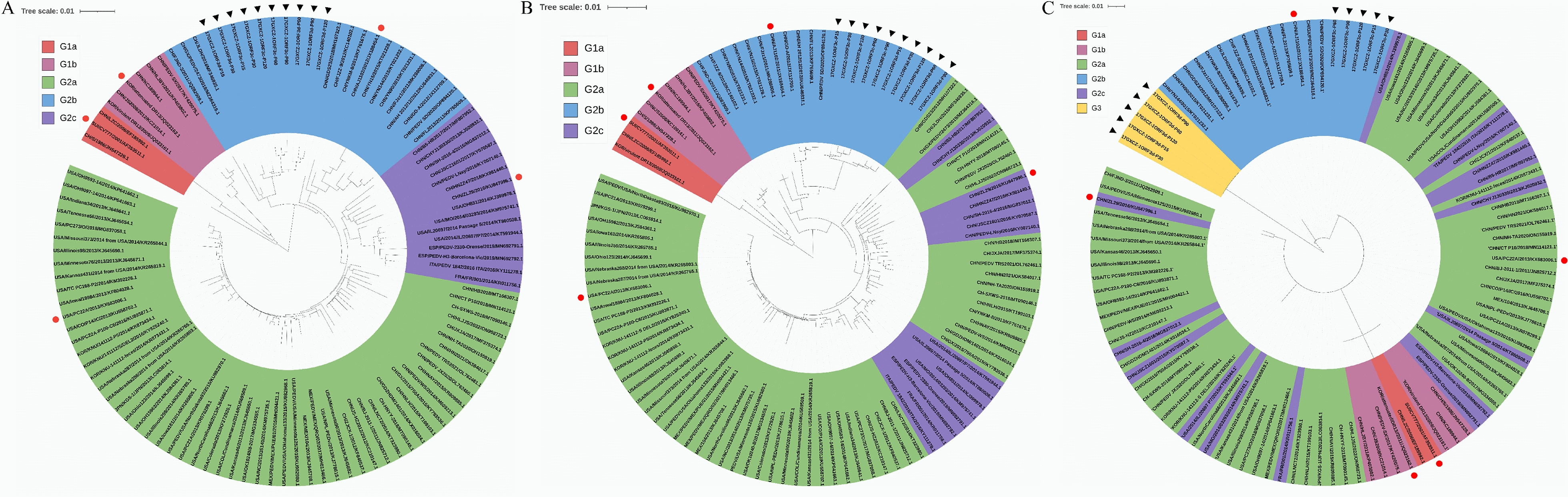
**Phylogenetic analyses of different generations of PEDV 17GXCZ-1ORF3d and 17GXCZ-1ORF3c variants on the basis of different genes**. Phylogenetic trees were constructed on the basis of the aligned S protein (**A**), S–N gene nucleotides (**B**) and ORF3 protein (**C**) via the neighbour-joining method of MEGA-X with 1000 bootstrap replicates. Scale bars represent the branch lengths measured by the number of substitutions per site. Each PEDV strain is indicated in the following format: country of origin (three letter codes: CHN, China, JPN, Japan, KOR, Korea, MEX, Mexico, SUI, Switzerland and USA, the United States)/strain name/year of sample collection (GenBank accession number). The subgroups G1a, G1b, G2a, G2b, G2c and Group G3 are coded in red, rose red, light green, sky blue, lavender and bright yellow, respectively. The triangular symbols represent the 17GXCZ-1ORF3d and 17GXCZ-1ORF3c variants in this study, and the circles represent the reference strains of each genotype.

**Figure 4:**
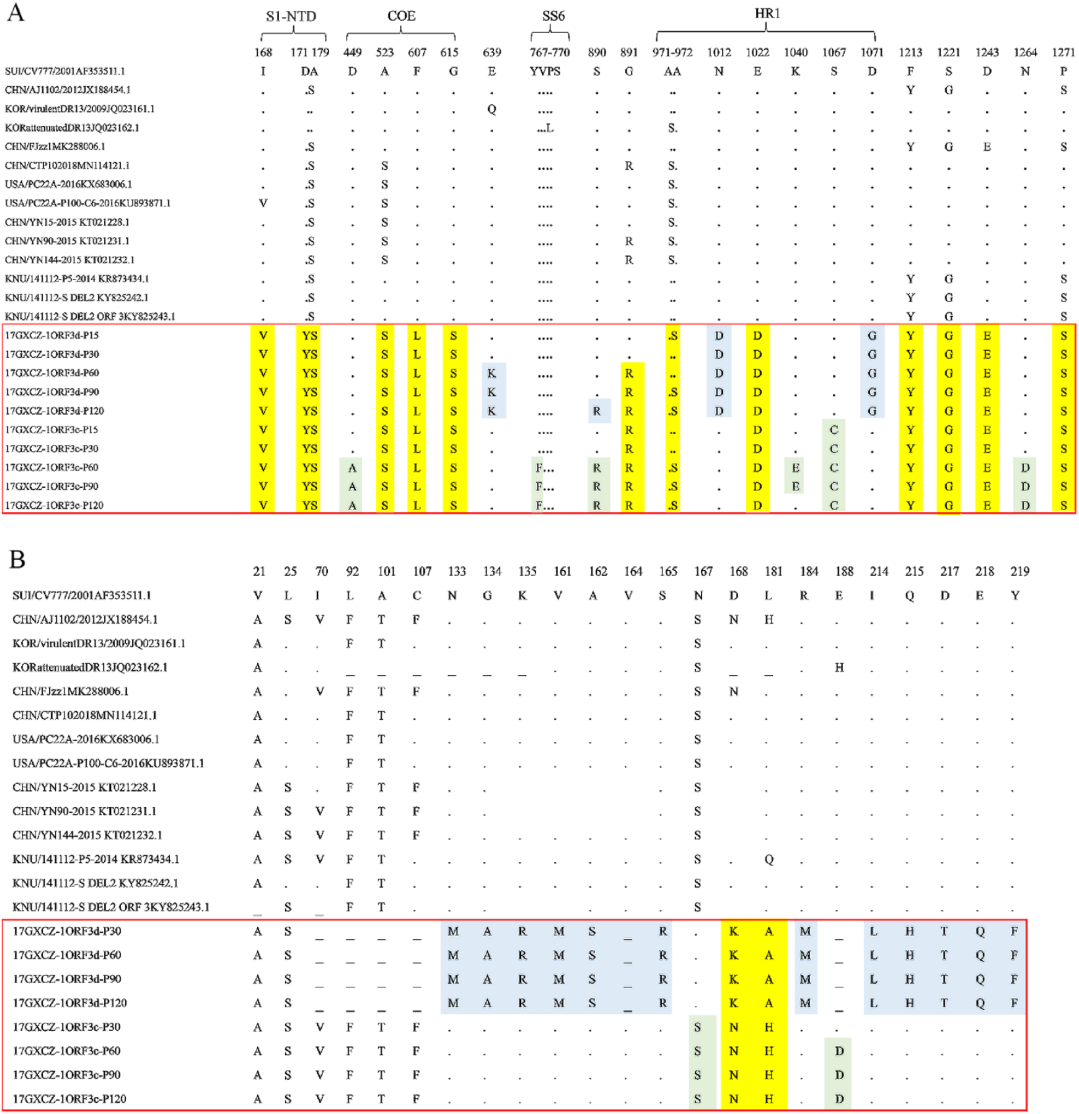
**Comparison of the S and ORF3 protein sequences of the PEDV 17GXCZ-1ORF3d and 17GXCZ-1ORF3c variants with those of other reference strains**. **A** Comparison of the S protein sequences of the PEDV 17GXCZ-1ORF3d and 17GXCZ-1ORF3c variants with those of other reference strains. The S protein aa sequences from the 17GXCZ-1ORF3d and 17GXCZ-1ORF3c variants (marked with red boxes) and other PEDV strains (including the classical CV777 strain) were compared with those of Clustal W. The S protein aa sequences of 17GXCZ-1ORF3d and 17GXCZ-1ORF3c were compared with those of other PEDV strains. **B** Comparison of the ORF3 protein sequences of the PEDV 17GXCZ-1ORF3d and 17GXCZ-1ORF3c variants with those of other reference PEDV strains. The aa sequences from the ORF3 proteins of 17GXCZ-1ORF3d and 17GXCZ-1ORF3c (marked by red boxes) and other PEDV strains (including the classical CV777 strain) were compared with those of ClustalW. The ORF3 protein aa sequences of 17GXCZ-1ORF3d and 17GXCZ-1ORF3c were compared with those of other PEDV strains. Among them, aa mutations that occur in both the 17GXCZ-1ORF3d and 17GXCZ-1ORF3c variants are highlighted in yellow, aa mutations specific to 17GXCZ-1ORF3d variants are highlighted in blue, and those specific to the 17GXCZ-1ORF3c variants are highlighted in green relative to the CV777 strain.

We compared the aa sequences of the S protein of the 17GXCZ-1ORF3d and 17GXCZ-1ORF3c variants with those of reference strains, including CV777 and AJ1102. The analysis revealed that both variants had relatively lower homologies of the S protein with CV777, ranging from 93.2 to 93.7% for 17GXCZ-1ORF3d and 92.9 to 93.7% for 17GXCZ-1ORF3c, respectively. Furthermore, both variants presented high homology to AJ1102, ranging from 97.7 to 98.3% for 17GXCZ-1ORF3d and 97.5 to 98.3% for 17GXCZ-1ORF3c (Additional file 2). In addition, a phylogenetic tree was constructed based on the S protein, S–N gene nucleotides and ORF3 protein of PEDV variants with the 107 reference strains found in GenBank [27] (Figure 3). The phylogenetic tree based on the S protein and S–N gene nucleotides revealed that the 17GXCZ-1ORF3d and 17GXCZ-1ORF3c variants belong to the G2b subgroup. Notably, with respect to the phylogenetic analysis based on the ORF3 protein, the 17GXCZ-1ORF3d variants were clustered into the G3 group [14, 28], as they shared aa homologies of 76.4 and 79.8% with the CV777 and AJ1102 strains, respectively (Additional file 2). However, attenuated DR13 and YN144, with early terminations in ORF3, were classified into subgroups G1b and G2b, respectively. After serial passage in vitro, compared with those of strain CV777, the 17GXCZ-1ORF3d and 17GXCZ-1ORF3c variants contained 4 aa insertions (^56^GENH^59^). However, the 17GXCZ-1ORF3d and 17GXCZ-1ORF3c variants in different generations presented two aa deletions in the S1-NTD region (^162^KDI^164^ → ^162^S△△^164^) relative to CV777. Among the 17GXCZ-1ORF3d and 17GXCZ-1ORF3c variants, three aa changes (I168V, D171Y and A179S) in the S1-NTD region, three aa changes (A523S, F607L and G615S) in the COE region of neutralizing epitopes, one aa change (E1022D) in the HR1 region, and four aa changes at sites F1213Y, S1221G, D1243E and P1271S (Figure 4A) were detected. Compared with those of the CV777 strain, a total of four aa changes (E639K, S890R, N1012D and D1071G) were observed in the S protein of the 17GXCZ-1ORF3d variants, whereas six aa changes (D449A, Y767F, S890R, K1040E, S1067C and N1264D) were observed in the 17GXCZ-1ORF3c variants. In addition, we compared the aa sequences of the ORF3 proteins from different generations of variants of these two strains. The 17GXCZ-1ORF3d variants had a long deletion in the nucleotides of the ORF3 gene, and translation was terminated early, whereas the 17GXCZ-1ORF3c variants had N167S, D168N and L181H changes, which were similar to those of AJ1102 (Figure 4B). This finding indicated that the genome sequence analysis and phylogenetic characterization of the 17GXCZ-1ORF3d and 17GXCZ-1ORF3c variants were altered during serial passaging in vitro.

### Pathogenicity of 17GXCZ-1ORF3d and 17GXCZ-1ORF3c via serial cell culture passages

**Figure 5:**
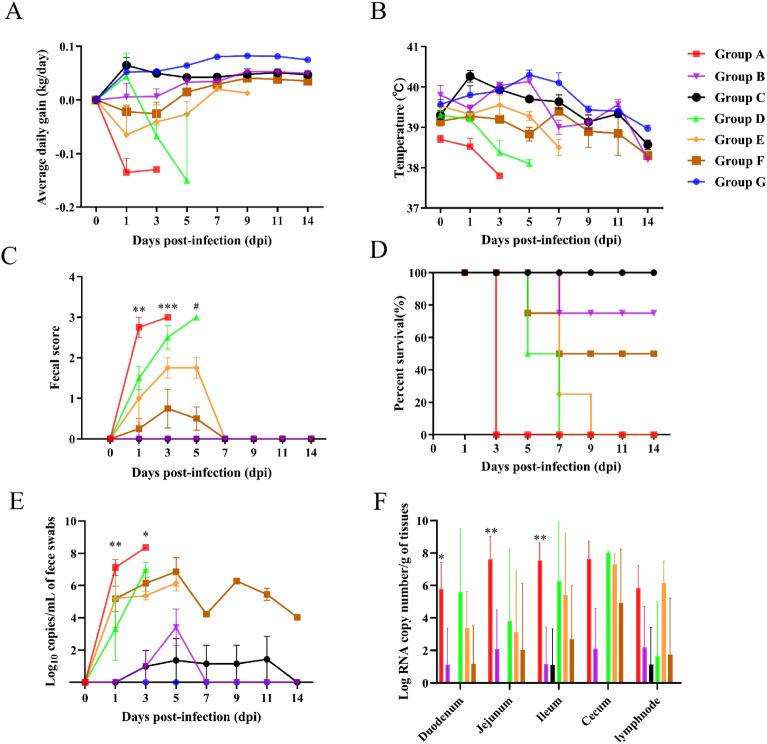
**Pathogenicity analysis of the PEDV 17GXCZ-1ORF3d and 17GXCZ-1ORF3c variants**. **A** Mean body weight changes in each group. **B** The average body weight changes in each group. **C** Fecal scores of the different groups of piglets after PEDV/mock infection. Rectal swabs were collected at different time points after PEDV infection and scored on the following criteria: 0, normal; 1, pasty stool; 2, semiliquid diarrhea; and 3, liquid diarrhea. **D** Survival rate of piglets in each group. **E** Viral RNA shedding from piglet feces after different numbers of PEDV/mock infection passages. **F** RNA copy number in different tissues of PEDV/mock-infected piglets. The asterisks indicate significant differences between 17GXCZ-1ORF3d-P15 and 17GXCZ-1ORF3d-P120 (*P < 0.05; **P < 0.01 and ***P < 0.001). The pound signs indicate significant differences between the 17GXCZ-1ORF3c-P15 and 17GXCZ-1ORF3c-P120 strains (^#^*P* < 0.05).

To determine the reason for PEDV strains 17GXCZ-1ORF3d and 17GXCZ-1ORF3c attenuation via serial cell culture passages, we used twenty-seven sucking piglets, which were divided into seven groups. The body weight and body temperature of each piglet were monitored daily. After PEDV inoculation, the body weights of the piglets in Groups A and D decreased within 3 dpi, whereas those of the piglets in Groups B, C and G increased. Additionally, the animals in Groups E and F did not grow all the time, although the overall trend was upwards (Figure 5A). The body temperature significantly decreased only when the animals died, and some piglets had temperatures exceeding 40 °C; however, this temperature rapidly returned to normal, and most of the remaining piglets remained within the normal range (Figure 5B). Piglets in Groups A and D experienced violent vomiting and diarrhea at 1 dpi and reached the peak of diarrhea at 3 dpi, and piglets in Groups E and F started to experience mild diarrhea at 1 dpi, with diarrhea peaking at 3 dpi. However, the piglets in Groups B, C and G were normal (Figure 5C).

In this study, the mortality rate of 5-day-old piglets in Groups A, D and E reached 100%, confirming that these strains are highly virulent (Table 3). Piglets in Group A gradually became moribund and died or were euthanized at 3 dpi (Figure 5D). One piglet in Group B was on the verge of death at 7 dpi, with a mortality rate of 25% (1/4), and the mortality rate of Group F reached 50% (2/4), where as all the piglets in Groups C and G survived. In conclusion, these data indicated that 17GXCZ-1ORF3d-P120 expression was attenuated with serial passages in vitro.

**Table 3:** **Pathogenicity evaluation of the 17GXCZ-1ORF3d and 17GXCZ-1ORF3c variants**

Groups	Inoculum dose (PFU/mL)	Mortality	Fecal shedding, log_10_ copies/mL, by PID, mean							Onset of clinical signs as judged by PIH^a^	Quantification of Viral load, log_10_ copies/mL, mean			PEDV antigen detection in frozen tissues^b^		
			1	3	5	7	9	11	14		D	J	I	D	J	I
17GXCZ-1ORF3d-P15 (Group A)	1.0 × 10^6^ (2 mL)	100%, 4/4	7.12	8.37	/	/	/	/	/	14–36	5.79	7.62	7.54	+	+ + +	+ + +
17GXCZ-1ORF3d-P90 (Group B)	1.0 × 10^6^ (2 mL)	25%, 1/4	-	0.99	3.42	–	–	–	–	120–144	1.13	2.08	1.15	–	–	–
17GXCZ-1ORF3d-P120 (Group C)	1.0 × 10^6^ (2 mL)	0%, 0/4	-	0.99	1.36	1.16	1.15	1.43	-	–	–	–	1.11	–	–	–
17GXCZ-1ORF3c-P15 (Group D)	1.0 × 10^6^ (2 mL)	100%, 4/4	3.31	6.98	/	/	/	/	/	18–36	5.62	3.82	6.27	–	+ +	+ + +
17GXCZ-1ORF3c-P90 (Group E)	1.0 × 10^6^ (2 mL)	100%, 4/4	5.20	5.38	6.15	/	/	/	/	30–54	3.38	3.12	5.42	–	+ +	+
17GXCZ-1ORF3c-P120 (Group F)	1.0 × 10^6^ (2 mL)	50%, 2/4	5.19	6.15	6.86	4.23	6.28	5.46	4.04	24–120	1.17	2.04	2.68	–	–	+ + +
Mock (Group G)	GM (2 mL)	0%, 0/4	–	–	–	–	–	–	–	–	–	–	–	–	–	–

^a^PIH: post-inoculation hour; D: duodenum; J: jejunum; I: ileum; PFU: plaque formation unit; –: no result.

^b^PEDV antigen detection in frozen tissues. + ,  +  + , and +  +  + denote less than 30%, 30–60% and more than 60% of villous enterocytes, respectively, showing a PEDV antigen-positive signal. The viral loads in fecal waste and in the tissues of the small intestine were detected via RT-qPCR.

### Viral shedding in feces and viral loads in different intestinal segments

Virus shedding from feces was investigated by RT-qPCR (Figure 5E). The piglets in Group A experienced faecal shedding within 1 dpi and reached the highest level of virus shedding within 3 dpi, with an average titre of 8.37 log_10_ copies/mL. Piglets in Group B were found to shed fecal viruses at 3 dpi and reached their highest excretion of 3.42 log_10_ copies/mL at 5 dpi, whereas those in Group C started excreting fecal viruses at 3 dpi, with a small amount of excretion ranging from 0.99 to 1.43 log_10_ copies/mL. The piglets in Group D experienced fecal shedding within 1 dpi and reached the highest level of virus shedding within 3 dpi, with an average titre of 6.98 log_10_ copies/mL. Compared with the animals in Group E, those in Group F experienced faecal shedding at 1 dpi and reached the highest level of 6.86 log_10_ copies/mL (Table 3).

We simultaneously examined the viral loads in different segments of the intestines, including the duodenum, jejunum, ileum, cecum, mesenteric lymph nodes (MLNs) and stomach (Figure 5F). As shown in Table 3, the segments of the duodenum, jejunum and ileum presented high viral loads in Group A, ranging from 5.79 to 7.62 log_10_ copies/g, whereas the viral load was detected only in the ileum of piglets in Group C, with a low value of 1.11 log_10_ copies/g. This value was significantly lower than the amount observed in the piglets of Group A (*P* < 0.01). The viral loads in the duodenum, jejunum and ileum of piglets in Group B ranged from 1.13 to 2.08 log_10_ copies/g. For the 17GXCZ-1ORF3c variants, piglets in Group F presented lower virus shedding and viral loads than those in Groups D and E did. Neither virus shedding in the feces nor the viral load in the tissues was detected in the mock group of piglets. Therefore, the pathogenicity of 17GXCZ-1ORF3d-P120 was significantly attenuated in piglets compared with that of 17GXCZ-1ORF3d-P15.

### Histopathological lesions in piglets infected with the 17GXCZ-1ORF3d and 17GXCZ-1ORF3c variants

**Figure 6:**
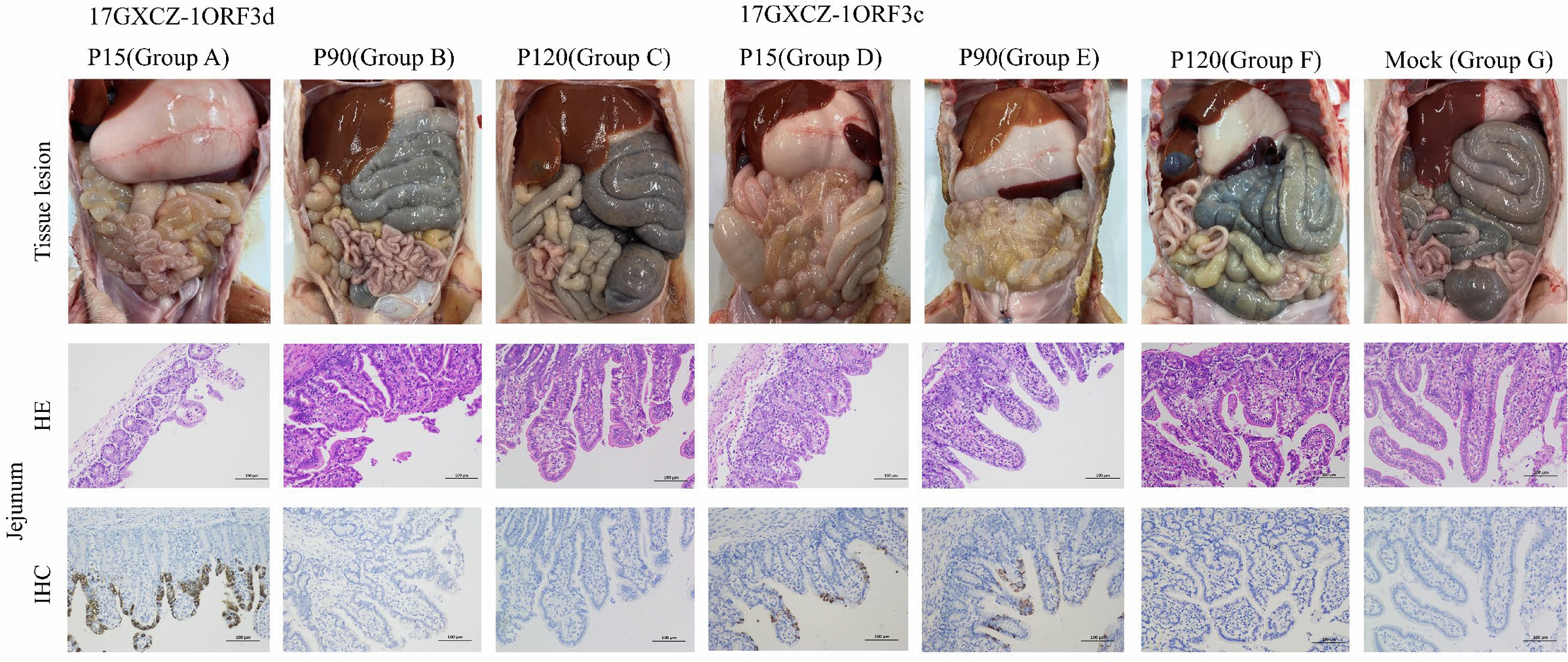
**Clinical autopsy of the intestinal tract of seven groups of piglets**. PEDV 17GXCZ-1ORF3d and 17GXCZ-1ORF3c variants as well as the small intestines of representative pigs from negative control animals were examined for gross lesions. Histopathology of the intestines of piglets inoculated with the PEDV 17GXCZ-1ORF3d and 17GXCZ-1ORF3c variants. H&E staining (200 ×) of the jejunum of piglets inoculated with 17GXCZ-1ORF3d and 17GXCZ-1ORF3c variants. IHC staining of the jejunum of piglets inoculated with 17GXCZ-1ORF3d and 17GXCZ-1ORF3c variants (200 ×). The PEDV antigen showed brown staining and was detected in the jejunal epithelial cells of low-generation virus-infected piglets infected with 17GXCZ-1ORF3d and 17GXCZ-1ORF3c. Intestinal tissue sections were stained with an anti-PEDV S protein monoclonal antibody and then incubated with an HRP-conjugated goat anti-mouse antibody, followed by fluorescence microscopy.

In this study, the piglets were necropsied once they reached the humanitarian endpoint. The remaining animals in each group were euthanized at the end of the study for postmortem assessment (Figure 6). All piglets in Groups A and D were found to exhibit typical PEDV lesions in their intestines after autopsy. The intestinal walls were thin or even transparent, with a large amount of yellowish fluid in the intestinal cavity. The jejunum of infected piglets harbouring the 17GXCZ-1ORF3d and 17GXCZ-1ORF3c variants were stained with H&E, and Groups A and D exhibited shortening, atrophy and even loss of intestinal villi. However, Groups B, E and F presented only slight damage to the intestinal villi (Figure 6). Piglets in Group C presented no significant pathological changes in either anatomy or histopathology and appeared to be similar to those in the mock group.

After the piglets were euthanized, serial sections of the duodenum, jejunum and ileum from all the groups were subjected to PEDV-specific IHC (Figure 6). In Group A, positive signals for PEDV antigen were detected in the duodenum (1–30%), jejunum (60–100%) and ileum (60–100%), whereas no signals were detected in Groups B and C (Table 3). The jejunum (30–60%) and ileum (60–100%) of Group D, the jejunum (30–60%) and ileum (1–30%) of Group E and the ileum (60–100%) of Group F presented PEDV antigen signals. None of the four piglets in the mock group showed any signs of positivity after IHC. These results demonstrated that the pathogenicity of 17GXCZ-1ORF3d-P120 was significantly lower than that of 17GXCZ-1ORF3d-P15.

### Passive protection against PEDV in piglets is conferred by colostrum from sows immunized with the 17GXCZ-1ORF3d-P120 vaccine candidate

**Figure 7:**
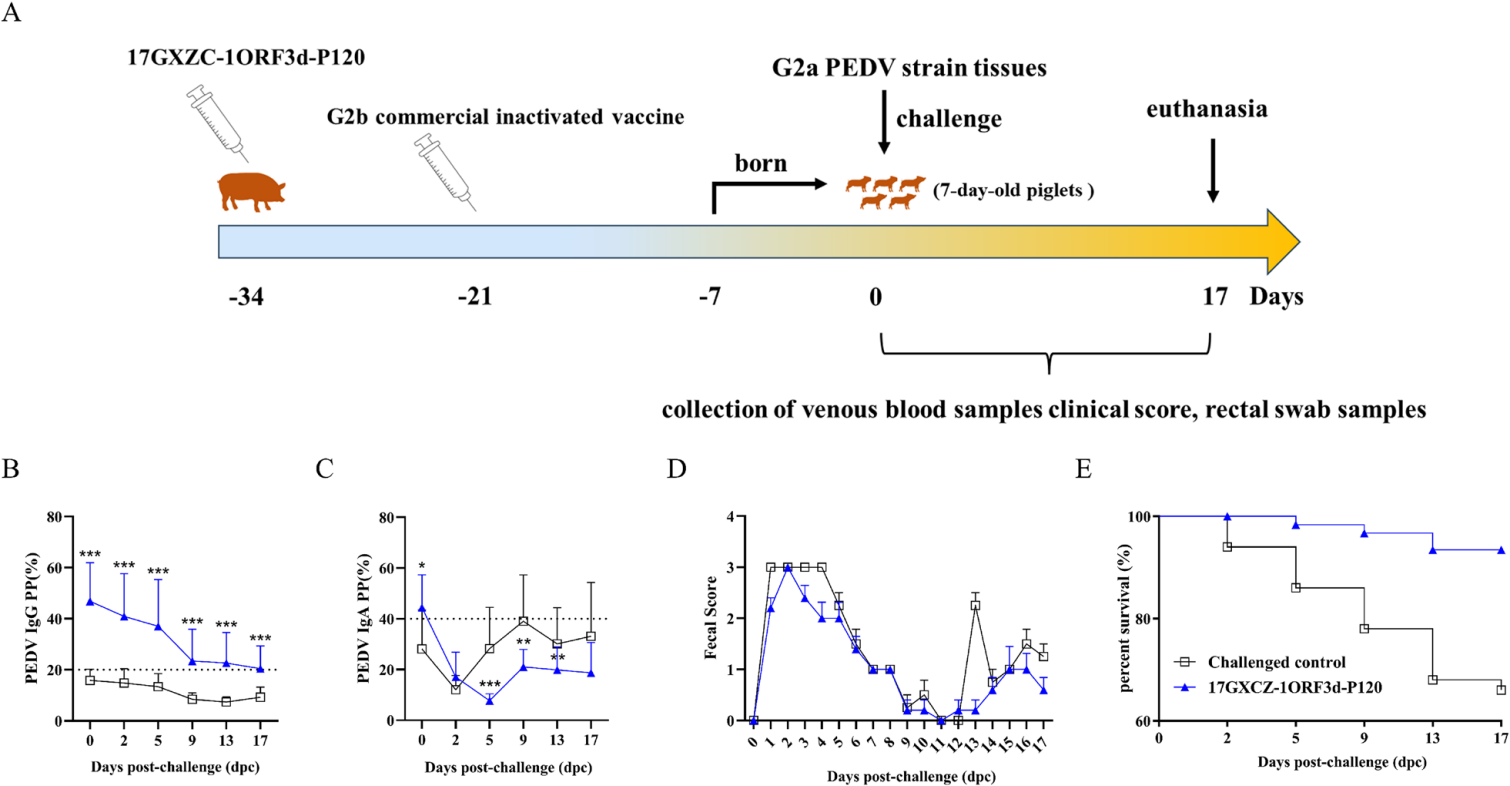
**Passive protection against PEDV in piglets can be provided by colostrum from immunized sows**. **A** Immunization procedures for sows and sample collection from piglets. Piglets born to sows immunized with the 17GXCZ-1ORF3d-P120 vaccine candidate and those born to unimmunized sows were infected with the PEDV strain. Serum samples were collected at 0, 2, 5, 9, 13 and 17 dpc to determine the levels of PEDV-N-protein-specific IgG (**B**) and PEDV-S-protein IgA (**C**) against the PEDV strain (*P < 0.05; **P < 0.01 and ***P < 0.001). Faecal scores (**D**) of the faeces after challenge with the PEDV strain. **E** Survival rates of piglets in the 17GXCZ-1ORF3d-P120/challenged control groups.

The ability of the live G2b 17GXCZ-1ORF3d-P120 vaccine candidate to confer passive immunity and protection was evaluated in piglets. After suckling for 7 days, the piglets from immunized and control sows were challenged with G2a PEDV homogenized intestinal tissues (Figure 7A). The levels of IgG and IgA were measured at 0, 2, 5, 9, 13 and 17 dpc in piglets from the 17GXCZ-1ORF3d-P120 group and the challenged control group (Figures 7B and C). Piglets in the 17GXCZ-1ORF3d-P120 group produced high levels of IgG, which were significantly higher than those in the challenged control group. Moreover, the IgA levels of piglets in the 17GXCZ-1ORF3d-P120 group were significantly higher at 0 dpc but decreased rapidly at 2 dpc and were significantly lower than those of the challenged control group at 5, 9 and 13 dpc. Although the fecal scores of piglets in the 17GXCZ-1ORF3d-P120 group were comparable to those of the challenged control group (Figure 7D), the total survival rate of piglets in the 17GXCZ-1ORF3d-P120 group was 93.44% (57/61), which was 27.44% greater than that of the challenged control group (Figure 7E). Compared with those in the 17GXCZ-1ORF3d-P120 group, the piglets in the control group had thin-walled intestines and yellow watery fecal contents at necropsy (Additional file 3). The viral loads in the stomach, lymph nodes, jejunum, ileum, and rectum of the 17GXCZ-1ORF3d-P120 group were similar to those in the challenged control group, but no viral loads were detected in the spleen, liver, lungs, duodenum, or cecum in the 17GXCZ-1ORF3d-P120 group (Additional file 3). However, the truncated ORF3 gene was not detected in the jejunal tissues of the piglets via RT-PCR (data not shown), indicating the safety of the attenuated strain. Taken together, these results indicated that G2b-attenuated 17GXCZ-1ORF3d-P120 could provide partial protection against the G2a PEDV strain.

## Discussion

Diarrheal disease is a leading cause of neonatal piglet mortality, although bacteria such as *Escherichia coli* and *Clostridium perfringens* can also potentially cause pre- and post-weaning diarrhea [29]. Emerging/reemerging coronaviruses (CoVs) can cause acute gastroenteritis in neonatal piglets, and an emerging SADS-CoV/PEAV/SeACoV was reported in 2017, characterized by acute diarrhea in neonatal piglets [30–32]. In recent years, PED epidemics caused by G2 PEDV variants have caused severe dehydration and high mortality in piglets, resulting in considerable economic losses to the global pig industry [9]. Generally, passage of a virus in vitro results in attenuation during the process of adapting to new cell lines [20, 21]. To understand the genetic characteristics of the PEDV variants and their pathogenicity changes during serial passages in vitro, a highly virulent G2 strain, 17GXCZ-1ORF3d, with a naturally truncated ORF3 gene, was serially propagated for up to 120 passages. In this study, infection with 17GXCZ-1ORF3d-P15 resulted in the formation of small plaques in Vero cells, and the high-passage 17GXCZ-1ORF3d variants grew more efficiently and formed larger plaques, which was consistent with the findings of a previous study [14]. Moreover, the viral titres of the 17GXCZ-1ORF3d variants increased with each generation during serial passaging, suggesting that these variants had good viral replication ability in vitro. Taken together, these findings suggest that high passage of 17GXCZ-1ORF3d-P120 positively influences in vitro viral growth.

The PEDV S protein is located in the outer layer of the viral envelope and is associated with genetic variation, viral replication, pathogenicity and trypsin-dependent proliferation of the virus [16]. The S protein is divided into two parts: S1 (19–726 aa) and S2 (727–1383 aa) [33]. Neutralization epitopes are present in S proteins, including the COE (499–638 aa) on the S1 subunit and the SS2 (748–755 aa), SS6 (764–771 aa) and 2C10 (1368–1374 aa) epitopes on the S2 subunit. During the in vitro serial passaging of the two strains, aa mutations were present in the S protein of P120. Compared with those in low-passage P15, the variants (P30, P60, P90 and P120) in 17GXCZ-1ORF3d had 5, 8, 11 and 13 aa changes, respectively. In contrast, the variants (P30, P60, P90 and P120) in 17GXCZ-1ORF3c had 5, 12, 20 and 18 aa changes, respectively (Table 1). This finding indicated that 17GXCZ-1ORF3d is genetically more stable during in vitro serial passaging. Compared with the aa of the 17GXCZ-1ORF3d variants, the S27L mutation is located in the S1-NTD region and may be involved in receptor binding, promoting viral attachment [34]. The E636K mutation on the epitope COE of 17GXCZ-1ORF3d-P120 was identified, and it may be related to neutralizing antibodies [35]. Two aa mutations (S887R and G888R) in the S2 subunit may be closely related to trypsin dependency [21, 36]. An A969S mutation in the S2 subunit was found in 17GXCZ-1ORF3d-P120, and a similar mutation at this site was found in the attenuated PT-P96 strain [37]. These aa mutations may lead to attenuated virulence, which needs to be confirmed by further studies. Additionally, on the basis of the results of the animal experiments in this study, the aa mutations found in P90 and P120 of the 17GXCZ-1ORF3c strain may not be associated with pathogenicity. Our group previously evaluated the pathogenicity of strains 17GXCZ-1ORF3d and 17GXCZ-1ORF3c in 7-day-old piglets, and those inoculated with the former had a higher mortality rate (75% vs 50%) than did those inoculated with the latter [14]. In this study, we found that 5-day-old piglets infected with 17GXCZ-1ORF3d-P15 developed diarrhea earlier, caused more severe watery diarrhea, and had a high mortality (100%) rate, which is consistent with the findings of our previous study. This indicated that the mortality rate of piglets infected with PEDV may be related to the age of the infected animals. We confirmed that low-passage 17GXCZ-1ORF3d with a naturally truncated ORF3 gene was highly pathogenic. Although the piglets in the group receiving 17GXCZ-1ORF3d-P90 did not experience diarrhea or vomiting, one piglet was depressed, with appetite and weight loss and very low body temperature at 5 dpi. It reached the humanitarian endpoint with no PEDV-positive antigens detected, and similar results and endpoints were reported in a previous study [14]. In contrast, piglets in the group given 17GXCZ-1ORF3d-P120 presented no obvious clinical symptoms and had weight gain comparable to that of the mock group (Figure 6). This confirmed that high-passage 17GXCZ-1ORF3d-P120 expression was significantly attenuated after serial passage in vitro.

In this study, colostrum from 17GXCZ-1ORF3d-P120-immunized sows was evaluated to determine whether colostrum provides passive protection against PEDV in piglets. To ensure that each sow is immunized with the appropriate dose, the attenuated vaccine is administered to sows via the intramuscular route. The measurement of IgA levels in serum samples may be a marker of passive protection [23, 38, 39], and the IgA antibodies absorbed by piglets from sow colostrum provide protective immunity to piglets [35]. Compared with those in the challenged control group, piglets in the 17GXCZ-1ORF3d-P120 group produced high levels of PEDV-specific IgA antibodies at the early stage but decreased rapidly, suggesting that IgA was consumed against the challenge of PEDV, which might explain the improved survival of piglets in the 17GXCZ-1ORF3d-P120 group. The results of these studies indicated that vaccines provide protection against homologous PEDV challenges and provide variable protection against heterologous PEDV [40–42]. In this study, piglets in the G2b 17GXCZ-1ORF3d-P120 group exhibited partial protection against the PEDV G2a strain.

ORF3 is the only accessory gene of PEDV and may play a crucial role in viral virulence [43–45]. Deletion of ORF3 might affect its cellular localization and transport and consequently influence its intracellular interplay with cell-host as well as viral interactions [43]. The mutations and deletions that occurred in the ORF3 gene in serial in vitro passages of PEDV led to early termination of translation, and these strains were attenuated in vivo [13, 19, 20]. These authors reported that the cell-adapted S DEL2/ORF3 and S DEL5/ORF3 viruses were completely attenuated in vivo, suggesting that ORF3 deletions resulting from large numbers of deletions may alter the pathogenicity of PEDV in its natural host [19]. In the present study, the 17GXCZ-1ORF3c strain with an intact ORF3 gene was used as a normal control, and the incompletely attenuated 17GXCZ-1ORF3c-P120 was found to have three aa mutations in ORF3. However, the naturally truncated ORF3 gene in the attenuated 17GXCZ-1ORF3d-P120 strain could be stably inherited via serial passage in vitro, indicating that this gene might accelerate the attenuation of virulence. In addition to the naturally truncated ORF3 gene, aa mutations in other genes, such as the S gene, have also been found in attenuated viruses, and whether these mutations affect the pathogenicity of PEDV needs to be further investigated.

In conclusion, 17GXCZ-1ORF3d was serially propagated in cell cultures and characterized after 120 passages. The 17GXCZ-1ORF3d-P120 strain presented larger plaques and higher viral titres, indicating that the cellular adaptation of 17GXCZ-1ORF3d-P120 occurred through in vitro serial passaging. In piglets inoculated with 17GXCZ-1ORF3d-P120, fecal viral shedding and the viral load in the jejunum were significantly lower than those in those inoculated with 17GXCZ-1ORF3d-P15. No obvious histopathological lesions were observed in the piglets of the attenuated 17GXCZ-1ORF3d-P120 group. In this study, immunization with 17GXCZ-1ORF3d-P120 significantly reduced the mortality rate of piglets after the onset of PED. Additionally, the attenuated strain with the truncated ORF3 gene could be used as a live vaccine candidate to provide partial passive protection for nursing piglets. Further work should aim to investigate the genetic variation of other genes associated with virulence, particularly the S gene, and the role of the ORF3 protein in pathogenesis. Therefore, we speculated that the attenuated PEDV strain with a naturally truncated ORF3 gene may be a potential candidate for developing live attenuated vaccines.

## Supplementary Information

**Additional file 1**: **Information regarding the reference strains of PEDV**.

**Additional file 2**: **Homology analysis of the 17GXCZ-1ORF3d and 17GXCZ-1ORF3c variants**.

**Additional file 3**: **Viral shedding, gross lesions and viral loads in different tissues from the 17GXCZ-1ORF3d-P120/challenged control groups of piglets**. **A** Viral shedding in the faeces of piglets after challenge with the PEDV strain. **B** Gross lesions in the jejunum of intestines collected from the 17GXCZ-1ORF3d-P120-infected and -challenged control groups. **C** Viral loads in different tissues from the 17GXCZ-1ORF3d-P120-challenged control groups of piglets.

## Data Availability

The genome sequences of the PEDV 17GXCZ-1ORF3d and 17GXCZ-1ORF3c variants obtained in this study have been deposited in GenBank under the accession numbers OR365272–OR365279, respectively.
