# Supplementary material for: Genetic characteristics associated with the virulence of porcine epidemic diarrhea virus (PEDV) with a naturally occurring truncated ORF3 gene
Source: Vet Res. 2024 Sep 27;55:123. doi: 10.1186/s13567-024-01384-w (PMC11437794; doi:10.1186/s13567-024-01384-w)
Supplement: Supplementary file 1 — Additional file 1: Information regarding the reference strains of PEDV. [file 13567_2024_1384_MOESM1_ESM.docx]

**Additional file 1.** **Information regarding the reference strains of PEDV.**

| Virus strain | Country of origin | Collection date | Accession number |
| --- | --- | --- | --- |
| CH/S | China | 1986 | JN547228.1 |
| CV777 | Switzerland | 2001 | AF353511.1 |
| LZC | China | 2006 | EF185992.1 |
| JS2008 | China | 2008 | KC109141.1 |
| virulent DR13 | South Korea | 2009 | JQ023161.1 |
| attenuated DR13 | South Korea | 2011 | JQ023162.1 |
| CH/FJND-3/2011 | China | 2011 | JQ282909.1 |
| AJ1102 | China | 2011 | JX188454.1 |
| BJ-2011-1 | China | 2011 | JN825712.1 |
| FJzz1 | China | 2011 | MK288006.1 |
| HLJBY | China | 2011 | KP403802.1 |
| AH 2012/12 | China | 2012 | KU646831.1 |
| CH/FJZZ-9/2012 | China | 2012 | KC140102.1 |
| CH/ZJCX-1/2012 | China | 2012 | KF840537.1 |
| GD-A | China | 2012 | JX112709.1 |
| GDS23 | China | 2012 | MH107322.1 |
| JS-HZ2012 | China | 2012 | KC210147.1 |
| KC189944.1 | China | 2012 | KC189944.1 |
| CHYJ130330 | China | 2013 | KJ020932.1 |
| CH/YNKM-8/2013 | China | 2013 | KF761675.1 |
| FL2013 | China | 2013 | KP765609.1 |
| KGS-1/JPN/2013 | Japan | 2013 | LC063814.1 |
| NPL-PEDv/2013 | USA | 2013 | KJ778615.1 |
| PC21A | USA | 2013 | KR078299.1 |
| PC22A | USA | 2013 | KX683006.1 |
| USA/Colorado/2013 | USA | 2013 | KF272920.1 |
| USA/NorthCarolina66/2013 | USA | 2013 | KJ645662.1 |
| TC PC168-P2 | USA | 2013 | KM392226.1 |
| USA/Iowa/18984/2013 | USA | 2013 | KF804028.1 |
| CO/P14/IC | USA | 2013 | KU558702.1 |
| MEX/104/2013 | Mexico | 2013 | KJ645708.1 |
| USA/Indiana34/2013 | USA | 2013 | KJ645641.1 |
| USA/Minnesota76/2013 | USA | 2013 | KJ645671.1 |
| USA/NC/2013/35140 | USA | 2013 | KM975735.1 |
| USA/Tennesse56/2013 | USA | 2013 | KJ645654.1 |
| USA/Minnesota90/2013 | USA | 2013 | KJ645682.1 |
| USA/Illinois98/2013 | USA | 2013 | KJ645690.1 |
| USA/Kansas46/2013 | USA | 2013 | KJ645650.1 |
| OH15962 | USA | 2013 | KJ584361.1 |
| CH/GDZHDM/1401 | China | 2014 | KX016034.1 |
| LNCT2 | China | 2014 | KT323980.1 |
| PEDV-WS | China | 2014 | KM609213.1 |
| FR/001/2014 | France | 2014 | KR011756.1 |
| KNU-141112-feces | South Korea | 2014 | KR873431.1 |
| OH851 | USA | 2014 | KJ399978.1 |
| COL/Cundinamarca/2014 | USA | 2014 | KU569509.1 |
| OH8593-14 | USA | 2014 | KP641662.1 |
| USA/2014/IL/20697 P7 | USA | 2014 | KT591944.1 |
| USA/IL20697/2014 Passage 5 | USA | 2014 | KT860508.1 |
| USA/Illinois259/2014 | USA | 2014 | KR265785.1 |
| USA/Iowa161/2014 | USA | 2014 | KR265805.1 |
| USA/Kansas431/2014 from USA | USA | 2014 | KR265819.1 |
| OH9097-14 | USA | 2014 | KP641663.1 |
| USA/Nebraska288/2014 from USA | USA | 2014 | KR265803.1 |
| USA/Missouri373/2014 from USA | USA | 2014 | KR265844.1 |
| USA/MO/2014/03293 | USA | 2014 | KM975741.1 |
| USA/Nebraska287/2014 from USA | USA | 2014 | KR265765.1 |
| USA/Ohio123/2014 | USA | 2014 | KJ645699.1 |
| KNU-141112-P5 | South Korea | 2014 | KR873434.1 |
| CH/GX/2015/750A | China | 2015 | KY793536.1 |
| CH/HNAY/2015 | China | 2015 | KR809885.1 |
| CH/HNLH/2015 | China | 2015 | KT199103.1 |
| YN15 | China | 2015 | KT021228.1 |
| YN90 | China | 2015 | KT021231.1 |
| YN144 | China | 2015 | KT021232.1 |
| PC22A-P100-C6 | USA | 2015 | KU893871.1 |
| PEDV/USA/Minnesota125/2015 | USA | 2015 | KU982980.1 |
| PEDV/MEX/PUE/01/2015 | Mexico | 2015 | MH004421.1 |
| PEDV/USA/NorthDakota93/2015 | USA | 2015 | KU982970.1 |
| PEDV/USA/Oklahoma133/2015 | USA | 2015 | KU982968.1 |
| CH/HNYF/14 | China | 2015 | KP890336.1 |
| CH/HNQX-3/14 | China | 2015 | KR095279.1 |
| ZL29 | China | 2016 | KU847996.1 |
| CH/JLDH/2016 | China | 2016 | MF346935.1 |
| CH/HNZZ47/2016 | China | 2016 | KX981440.1 |
| CHN/SH-2016-4/2016 | China | 2016 | MG837012.1 |
| JSCZ1601 | China | 2016 | KY070587.1 |
| PEDV-LNsy | China | 2016 | KY007140.1 |
| PEDV 1842/2016 ITA | Italy | 2016 | KY111278.1 |
| KNU-141112-S DEL2 | South Korea | 2016 | KY825240.1 |
| KNU-141112-S DEL2/ORF3 | South Korea | 2016 | KY825241.1 |
| B5-HB2017 | China | 2017 | MF807952.1 |
| CH/JXJA/2017 | China | 2017 | MF375374.1 |
| PEDV-SX | China | 2017 | KY420075.1 |
| PEDV/MEX/QRO/02/2017 | Mexico | 2017 | MH013466.1 |
| PC273/O | USA | 2017 | MG837058.1 |
| USA/OK10240-8/2017 | USA | 2017 | MG334555.1 |
| HB2018 | China | 2018 | MT166307.1 |
| CT P10 | China | 2018 | MN114121.1 |
| CH-HNYY-2018 | China | 2018 | MT090145.1 |
| CH-SXWS-2018 | China | 2018 | MT090146.1 |
| CH/GX/PEDV/2473/2019 | China | 2019 | MZ364316.1 |
| PEDV-2330-Orense | Spain | 2019 | MN692791.1 |
| PEDV-H3-Barcelona-Vic | Spain | 2019 | MN692792.1 |
| PEDV JX2020 | China | 2020 | OL762460.1 |
| PEDV SD/2020 | China | 2020 | OP894120.1 |
| NH-TA2020 | China | 2020 | ON155919.1 |
| PEDV TRS2021 | China | 2021 | OL762461.1 |
| HN2021 | China | 2021 | OK584017.1 |
| CH/HLJJS/2022 | China | 2022 | ON968723.1 |
