# Supplementary material for: Genetic characteristics associated with the virulence of porcine epidemic diarrhea virus (PEDV) with a naturally occurring truncated ORF3 gene
Source: Vet Res. 2024 Sep 27;55:123. doi: 10.1186/s13567-024-01384-w (PMC11437794; doi:10.1186/s13567-024-01384-w)
Supplement: Supplementary file 2 — Additional file 2: Homology analysis of the 17GXCZ-1ORF3d and 17GXCZ-1ORF3c variants. [file 13567_2024_1384_MOESM2_ESM.docx]

**Additional file 2. Homology analysis of the 17GXCZ-1ORF3d and 17GXCZ-1ORF3c variants.**

| PEDV Strain | Gene | Percentage of amino acid identity (%) | | Gene | Percentage of amino acid identity (%) | |
| --- | --- | --- | --- | --- | --- | --- |
|  |  | CV777 | AJ1102 |  | CV777 | AJ1102 |
| 17GXCZ-1ORF3d-P15 | S | 93.7 | 98.3 | ORF3 | 76.4 | 79.8 |
| 17GXCZ-1ORF3d-P30 |  | 93.5 | 98.1 |  | 76.4 | 79.8 |
| 17GXCZ-1ORF3d-P60 |  | 93.3 | 97.9 |  | 76.4 | 79.8 |
| 17GXCZ-1ORF3d-P90 |  | 93.2 | 97.7 |  | 76.4 | 79.8 |
| 17GXCZ-1ORF3d-P120 |  | 93.3 | 97.8 |  | 76.4 | 79.8 |
| 17GXCZ-1ORF3c-P15 |  | 93.7 | 98.3 |  | 95.6 | 100 |
| 17GXCZ-1ORF3c-P30 |  | 93.6 | 98.1 |  | 95.1 | 99.6 |
| 17GXCZ-1ORF3c-P60 |  | 93.1 | 97.7 |  | 94.7 | 99.1 |
| 17GXCZ-1ORF3c-P90 |  | 92.9 | 97.5 |  | 95.1 | 99.6 |
| 17GXCZ-1ORF3c-P120 |  | 93.0 | 97.6 |  | 94.7 | 99.1 |
