# Supplementary figures and images for: Genetic characteristics associated with the virulence of porcine epidemic diarrhea virus (PEDV) with a naturally occurring truncated ORF3 gene
Source: Vet Res. 2024 Sep 27;55:123. doi: 10.1186/s13567-024-01384-w (PMC11437794; doi:10.1186/s13567-024-01384-w)

## Slide 1
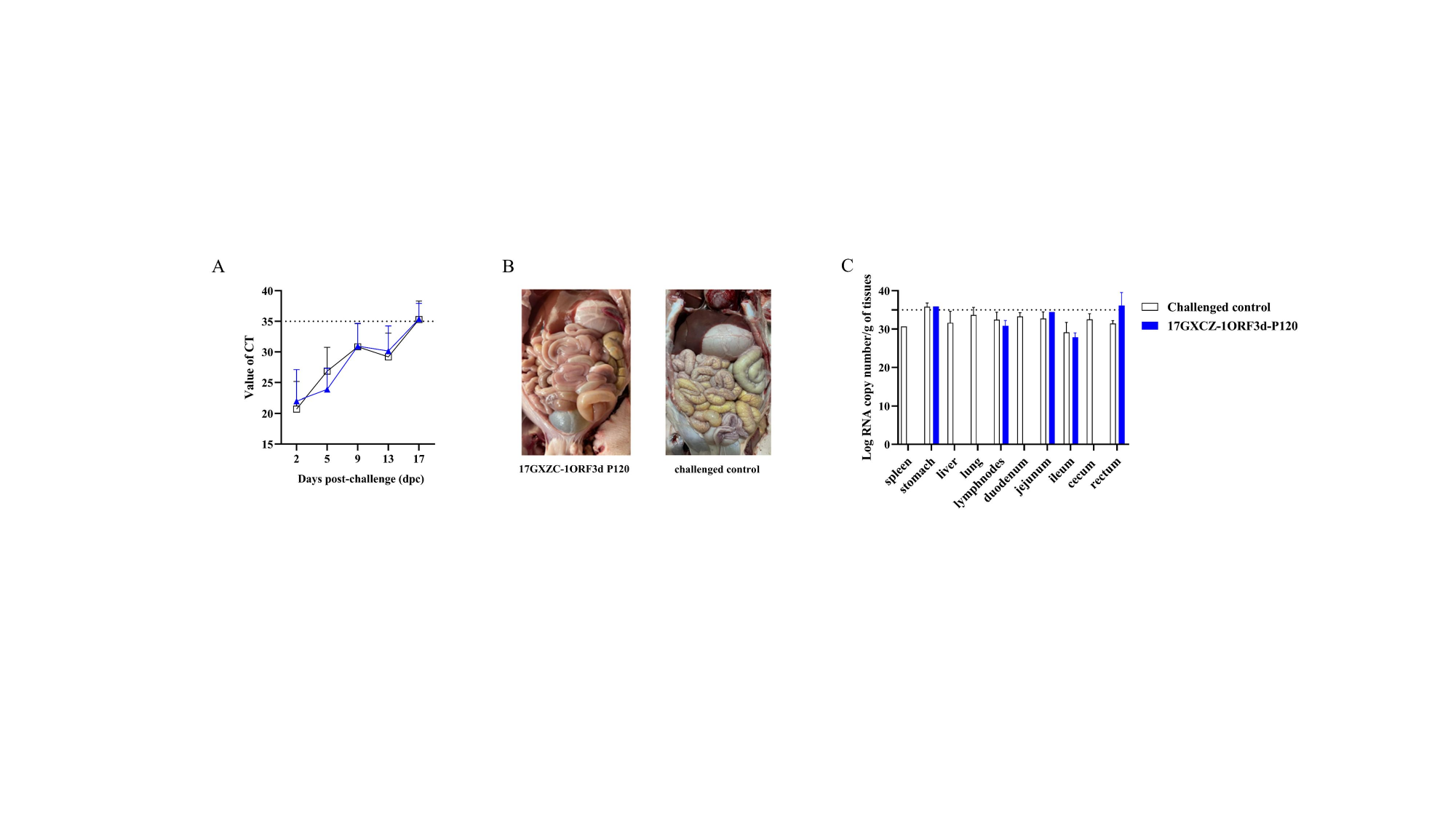

Supplement: Supplementary file 3 — Additional file 3: Viral shedding, gross lesions and viral loads in different tissues from the 17GXCZ-1ORF3d-P120/challenged control groups of piglets. A Viral shedding in the faeces of piglets after challenge with the PEDV strain. B Gross lesions in the jejunum of intestines collected from the 17GXCZ-1ORF3d-P120-infected and -challenged control groups. C Viral loads in different tissues from the 17GXCZ-1ORF3d-P120-challenged control groups of piglets. [file 13567_2024_1384_MOESM3_ESM.pptx]
